# Odontogenic cutaneous fistula mimicking malignancy

**DOI:** 10.1002/ccr3.907

**Published:** 2017-03-28

**Authors:** Akira Baba, Yumi Okuyama, Takeo Shibui, Hiroya Ojiri

**Affiliations:** ^1^ Department of RadiologyTokyo Dental College Ichikawa General HospitalChibaJapan; ^2^ Department of Oral Medicine, Oral and Maxillofacial SurgeryTokyo Dental CollegeChibaJapan; ^3^ Department of RadiologyThe Jikei University School of Medicine and University HospitalTokyoJapan

**Keywords:** Odontogenic cutaneous fistula, periodontal disease

## Abstract

It is important for the dentists to make accurate diagnosis and appropriate treatment of odontogenic cutaneous fistula. Although large facial skin lesions may bring up malignancy on top of the differential list, careful evaluation including physical observation, imaging, and pathology can rule out malignancies.

An 87‐year‐old man with a history of diabetes presented to our hospital due to dysphagia with skin perforation on his left chin. He was previously diagnosed with a traumatic hairline fracture of the mandible 2 weeks before. Observation revealed a 5‐cm ulcerated lesion in left submandibular region (Fig. 1) with purulent discharge. Vital signs were all normal. Laboratory investigation revealed inflammatory changes: WBC, 10,800/*μ*L; and C‐reactive protein, 2.23 mg/dL. Intraoral examination showed gingival swelling at the level of left impacted mandibular tooth. Computed tomography images revealed cutaneous ulcer at left submandibular area creating a fistula continuing from left third molar pericoronitis, with osteosclerosis (Fig. 2). Highly infiltrative morphology of ulceration indicated malignant possibility, and scraping cytology and biopsy were performed. Cytology was negative and biopsy showed various marked inflammatory cell infiltration including neutrophil without malignant potential. Clinical, radiological, and pathological findings led to the diagnosis of odontogenic cutaneous fistula. He underwent teeth extraction and oral antibiotics, the typical treatment of choice. The fistula subsided without complications. There has been no recurrent symptom for about 3 years. Odontogenic fistula is defined as pathologic communication between skin and oral cavity 1. It is rare and often misdiagnosed, leading to recurrence 1. The causes include caries, pulp infection, periapical/periodontal diseases, root fracture, and chemical/mechanical trauma 2. It must be differentiated from malignancies such as gingival cancer, osteosarcoma, Ewing's sarcoma, and giant cell tumor, as the treatment for these is complete resection sometimes followed by chemotherapy and/or radiation therapy. Odontogenic cutaneous fistula tends to be located on area of the mandible angle in relatively high frequency as with our case 3. It was reported the predominant morphological presentation is a nodule 3. However, the facial fistula in our case was so prominent, mimicking a malignancy. Careful planning of radiological, pathological, and intraoral evaluations eventually led us to the correct diagnosis. It is important for the dentists to make accurate diagnosis and appropriate treatment of odontogenic cutaneous fistula.

**Figure 1:**
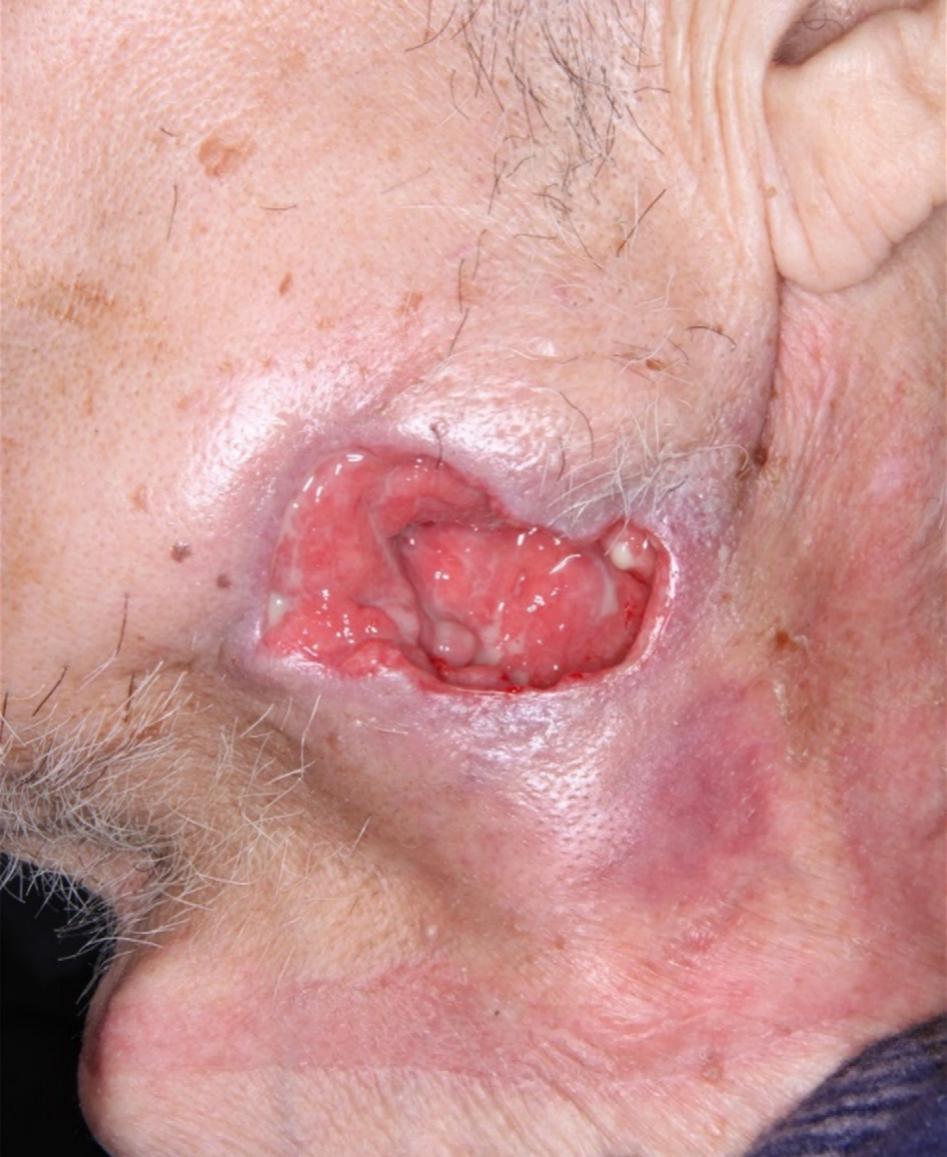
Observation revealed a cutaneous ulcerated lesion in left submandibular region.

**Figure 2:**
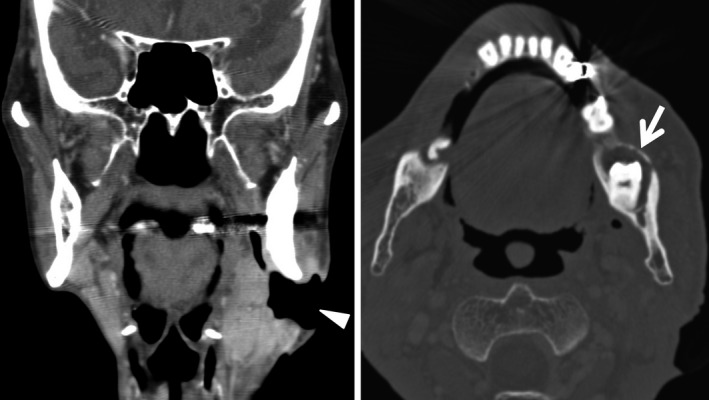
Contrast‐enhanced and bone window computed tomography images revealed cutaneous ulcer (arrow head) at left submandibular area creating a fistula continuous with left third molar pericoronitis with osteosclerosis (arrow).

## Authorship

AB: drafted the article. AB, YO, TS, and HO: participated in critical review and revision of the article, gave the final approval of the article, and have accountability for all aspects of the work.

## Conflict of Interest

None declared.
